# Examining comorbidities in children with diarrhea across four provinces of Mozambique: A cross-sectional study (2015 to 2019)

**DOI:** 10.1371/journal.pone.0292093

**Published:** 2023-09-26

**Authors:** Adilson Fernando Loforte Bauhofer, Júlia Sambo, Jorfélia J. Chilaúle, Carolina Conjo, Benilde Munlela, Assucênio Chissaque, Telma Isaías, Marlene Djedje, Nilsa de Deus

**Affiliations:** 1 Instituto Nacional de Saúde (INS), Distrito de Marracuene, Província de Maputo, Mozambique; 2 Instituto de Higiene e Medicina Tropical, Universidade Nova de Lisboa, Lisboa, Portugal; 3 Departamento de Ciências Biológicas, Universidade Eduardo Mondlane, Maputo Cidade, Mozambique; Clinton Health Access Initiative, UNITED STATES

## Abstract

Comorbidities are defined as the simultaneous occurrence of two or more diseases within the same individual. Comorbidities can delay a patient’s recovery and increase the costs of treatment. Assessing comorbidities can provide local health care policy-makers with evidence of the most common multi-health impairments in children. This could aid in redirecting and integrating care and treatment services by increasing health facilities the awareness and readiness of health facilities. The present analysis aims to determine the frequency and associated factors of comorbidities in children with diarrhea in Mozambique. A cross-sectional hospital-based analysis was conducted between January 2015 and December 2019 in children up to 59 months of age who were admitted with diarrhea in six reference hospitals in Mozambique. These hospitals are distributed across the country’s three regions, with at least one hospital in each province from each region. Sociodemographic and clinical data were obtained through semi-structured interviews and by reviewing the child clinical process. Descriptive statistics, and Mann-Whitney-U tests were used. Crude and adjusted logistics regression models were built. P-values < 0.05 were considered statistically significant. Comorbidities were observed in 55.5% of patients (389/701; 95%CI: 51.8–59.1). Wasting was the most common comorbidity (30.2%; 212/701) and pneumonia was the least common (1.7%; 12/701). Children born with a low birth weight were 2.420 times more likely to have comorbidities, adjusted odds ratio: 2.420 (95% CI: 1.339–4374). The median (interquartile range) duration of hospitalization was significantly higher in children with comorbidities than without comorbidities, 5 days (3–7) and 4 days (3–6), respectively (p-value < 0.001). One in every two children with diarrhea in Mozambique has an additional health impairment, and this increases the length of their hospital stay.

## Introduction

In 2019, lower respiratory infections, diarrheal diseases and malaria were among the main causes of death in children up to 59 months of age worldwide, accounting for 13.3%, 9.9%, and 7.1% of deaths, respectively [1]. Despite the decrease in total number of deaths from diarrheal diseases by 23.9%, from 2006 to 2016, diarrhea disease remains a public health concern, especially in children [1, 2].

Diseases such as pneumonia, HIV and undernutrition are pointed as risk factors for diarrhea, in children [3, 4]. Diarrhea associated with other diseases, can contribute to a higher morbidity and mortality rate [1, 5]. In African countries, comorbidities in children up to 59 months of age ranged from 2% to 11%, using Demographic Health Survey [6–10]. Other survey systems also reported the co-occurrence of acute respiratory infections, stunting and diarrhea [11]. Mozambique, an African country, reported increased risk of death in children with diarrhea that had either HIV, pneumonia or undernutrition [5].

Comorbidities can delay patient recovery and increase the costs of treatment. Assessing comorbidities can provide local health policy-makers with evidence of the most common multi-health impairments in children, and thus redirect and integrate care and treatment services by increasing the awareness and readiness of health facilities to the most common health impairment. Although it is recognized that certain conditions such as undernutrition, HIV, pneumonia, and malaria often coexist with diarrhea, particularly in low-and middle-income countries [12–16], precise and reliable estimates of frequency and impact of these are lacking. The present study aims to determine the frequency and associated factors of comorbidities in children up to 59 months of age with diarrhea in six hospitals in four provinces of Mozambique.

## Methods

### Study design, site, population

A cross-sectional hospital-based analysis was conducted between January 2015 to December 2019 in children admitted to six sentinel sites in Mozambique and captured through a diarrhea surveillance system. In the southern region of the country, the analysis included Hospital Central de Maputo, Hospital Geral José Macamo and Hospital Geral de Mavalane in Maputo city, in the center region it included Hospital Central da Beira in Sofala province and Hospital Geral de Quelimane in Zambezia province, and in the northern region included Hospital Central de Nampula in Nampula province.

A purposive sample was considered, eligible population for the present analysis were children up to 59 months of age admitted in the sentinel sites with diarrhea as the main reason for seeking health services, with known information for malaria, pneumonia, HIV, wasting and stunting and whose caregivers consented with their inclusion in the study. Diarrhea was defined as the passage of three or more loose or liquid stools in 24 hours [17].

### Sample size calculation

Due to the unknown prevalence of comorbidities in children with diarrhea in Mozambique, the 50% chance was used to estimate the sample size, considering a 5% desired precision, with a 95% confidence interval (CI), design effect of 1 and 80% power, the minimum sample size required was 384.

### Data collection

Sociodemographic and clinical data were obtained at the health facilities using semi-structured forms. Recorded data included information on the child’s sex, age, feeding habits, HIV status, birth weight and length in days of hospital stay. From the child’s mother, information on educational level and HIV status was collected.

### Sample collection and laboratory diagnosis

For each child, a single stool sample was collected and stored at minus 20°C for sentinel sites outside Maputo city and shipped to Instituto Nacional de Saúde (INS)–Mozambique. Samples from children in Maputo city were delivered to INS within a day of collection [18].

Collected samples were screened for Group A Rotavirus antigens using the commercial enzyme-immuno-sorbent assay kit (Prospect, Oxoid Ltd, United Kingdom), according to the commercial instructions. Intestinal parasites were screened by concentrating the stool samples using the formalin-ether technique and the modified Ziehl-Neelsen technique was used to identify coccidian by optical microscopy [19].

### Data management and statistical analysis

Data collected was double entered in Epi Info 3.5.1 (Center for Disease Control and Prevention, Atlanta, 2008) followed by data comparison and inconsistencies resolution. Comorbidity (dependent variable) was defined as the presence of pneumonia, malaria, HIV, wasting or stunting, in addition to diarrhea. Wasting and stunting status were assessed using the WHO software Anthro version 3.2.2 [20]. Pneumonia, malaria, and HIV status were based on the information presented in the child’s hospital record.

Data analysis was conducted in R, version 4.1.0 (Vienna, Austria) [21], and p-values < 0.05 were considered significant. Descriptive statistics were used to describe sample characteristics, including missing data. Relative frequency and Jeffreys 95% CI were computed for comorbidities, pneumonia, malaria, HIV, wasting and stunting status. Crude logistics regression models were built for each independent variable, the ones with p-values less than 10% were included in the adjusted logistic regression models. Pneumonia, malaria, HIV and wasting/stunting models results are in S1–S4 Tables, respectively. A Mann-Whitney-U test was used to compare the median length in days of hospital stay between children with and without comorbidities. The discharge probability curve was estimated and the difference by comorbidity status was assessed using the log-rank test.

### Ethical statement

Study protocol was approved by the National Bioethics Committee for Health in Mozambique (IRB00002657, reference 348/CNBS/13). Authors had access to information that could identify individual participants during or after data collection. Children were only enrolled after the written consent of their legal guardians was obtained.

## Results

### Sample characteristics

**Fig 1:**
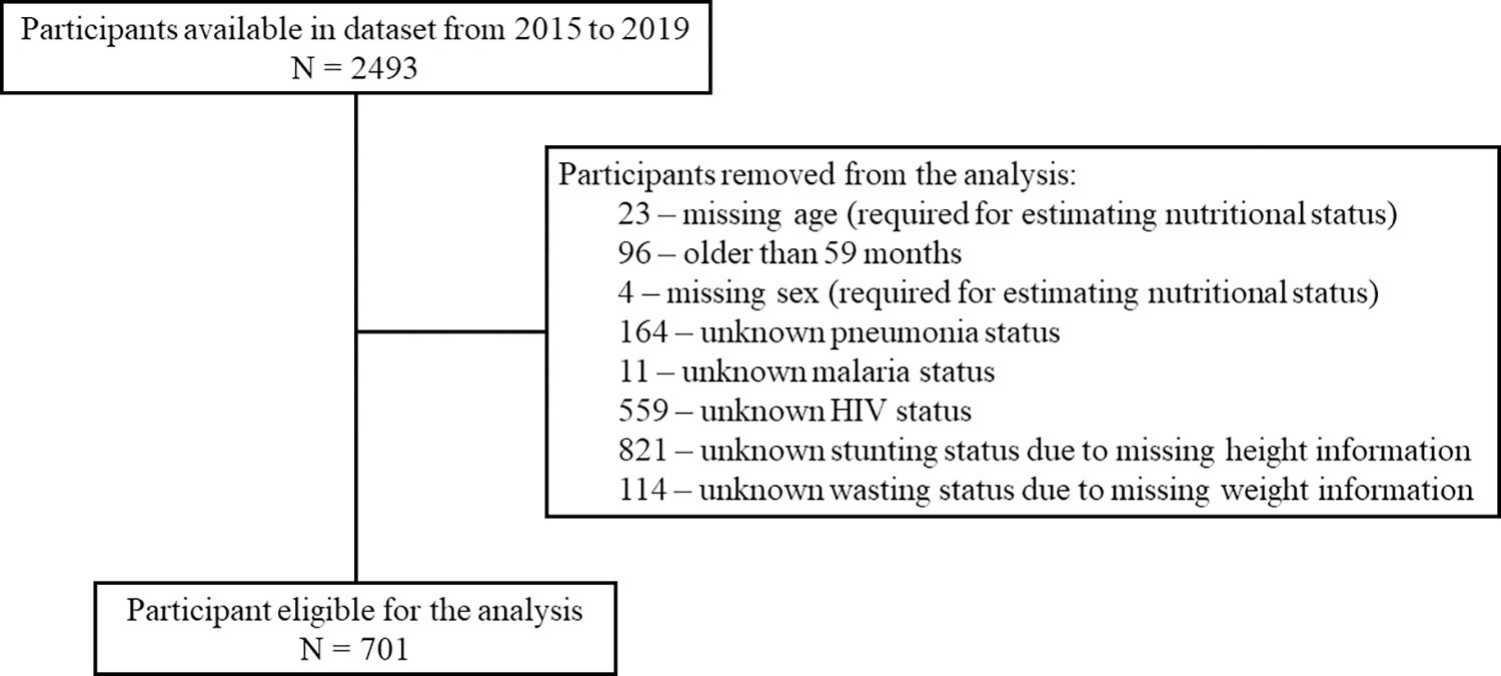
Available participants in the dataset and criteria for removal from the analysis.

From January 2015 to December 2019, there were 2493 cases of children with diarrhea captured through the hospital-based surveillance system. Of those, 1792 were not included in the analysis due to reasons such as: participants without age information (n = 23), older than 59 months (n = 96), without required information for estimating nutritional status, such as sex (n = 4), height (n = 821) and weight (n = 114), and participants without pneumonia (n = 164), malaria (n = 11) and HIV information (n = 559). In total, 701 children complied with the eligibility criteria (Fig 1).

Of the included participants, 58.9% (413/701) were males, 70.0% (491/701) were from hospital sites in Maputo city, 51.1% (355/701) had mothers with secondary/above education level, 24.1% (165/686) were exclusively breastfeed and 14.8% (92/622) were low birth weighted (Table 1).

**Table 1:** Sociodemographic and clinical characteristics of children with diarrhea, January 2015 to December 2019.

Characteristic (N = 701)	% (n)
**Sex**	
Male	58.9% (413)
Female	41.1% (288)
**Age in months (categorized)**	
0–11	41.7% (292)
12–23	40.8% (286)
24–59	17.5% (123)
**Province**	
Maputo city	70.0% (491)
Sofala	10.0% (70)
Zambezia	7.0% (49)
Nampula	13.0% (91)
**Mother’s education level**	
None	9.5% (66)
Primary	39.4% (274)
Secondary/above	51.1% (355)
Unknown	6
**Exclusive breastfeeding**	
No	75.9% (521)
Yes	24.1% (165)
Unknown	15
**Year**	
2015	10.8% (76)
2016	15.4% (108)
2017	30.8% (216)
2018	24.8% (174)
2019	18.1% (127)
** Low birth weight (< 2500 grams)**	
No	85.2% (530)
Yes	14.8% (92)
Unknown	79
** Child previously hospitalized due to diarrhea**	
No	88.8% (531)
Yes	11.2% (67)
Unknown	103
** Mother’s HIV status**	
No	70.6% (464)
Yes	29.4% (193)
Unknown	44

### Comorbidities and associated factors

**Fig 2:**
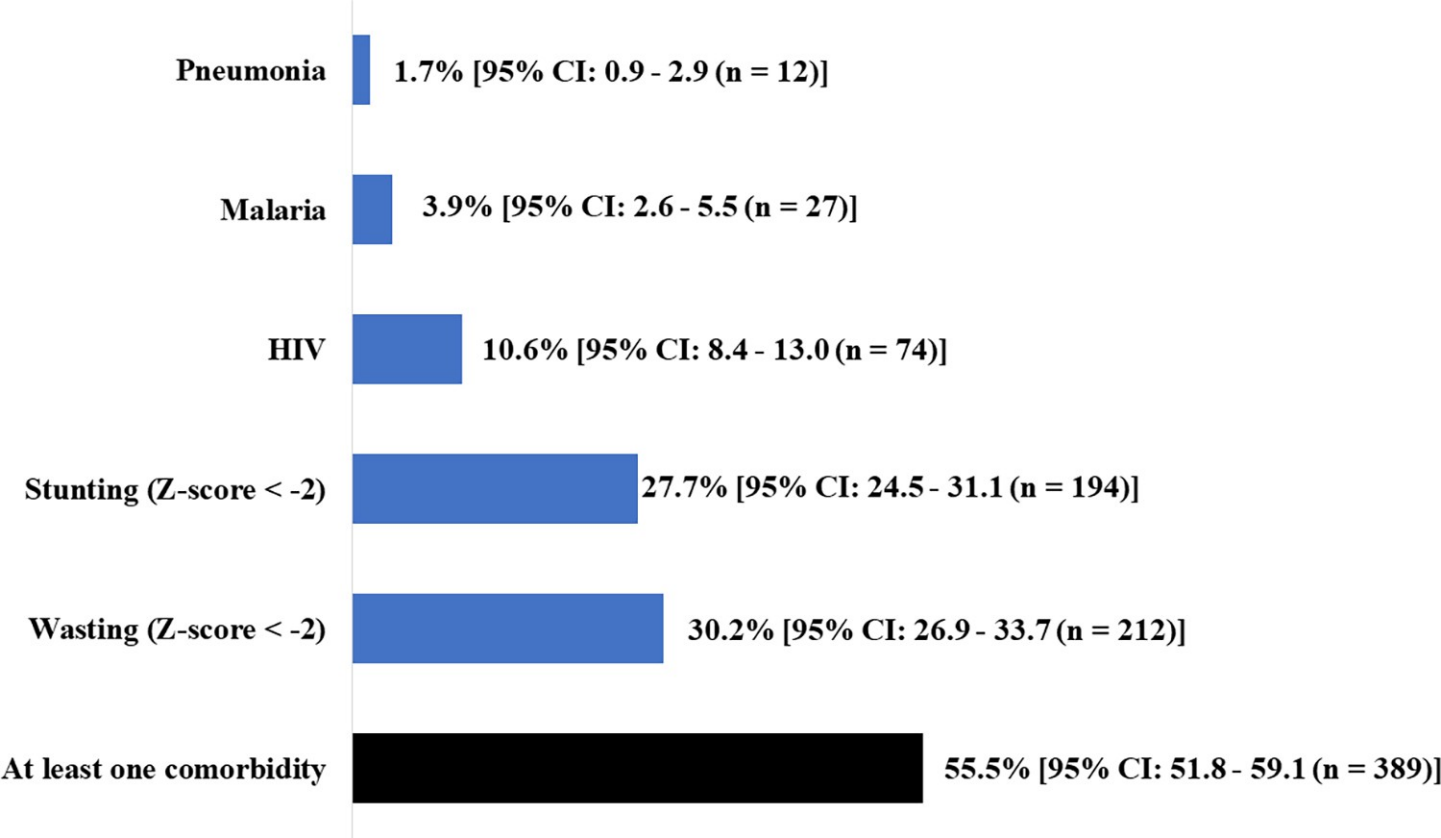
Overall comorbidity frequency and type of associated disease, January 2015 to December 2019 (N = 701).

Comorbidities were observed in 55.5% (95% CI: 51.8–59.1; 389/701). Wasting was the most common comorbidity (30.2%; 95% CI: 26.9–33.7; 212/701) and pneumonia was the least common (1.7%; 95% CI: 0.9–2.9; 12/701) (Fig 2).

Comorbidities were higher in children recruited in Nampula province (78.0%, 71/91), compared to the ones from Maputo city (47.3%; 232/491), adjusted odds ratio: 3.822 (95% CI: 2.085–7.007, p-value < 0.001). Comorbidities were 0.180 times (95% CI: 0.070–0.462, p-value < 0.001) less likely observed in children whose mothers had primary educational level (54.7%, 150/274) compared to mothers without an education level (89.4%, 59/66, Table 2). Children born with low birth weight were 2.420 times more likely of having comorbidities, adjusted odds ratio: 2.420 (95% CI: 1.339–4374) (Table 2).

**Table 2:** Sociodemographic and clinical characteristics and factors associated with comorbidities in children with diarrhea, January 2015 to December 2019.

Characteristics	% (n/N)	COR (95% CI)	p-value	AOR (95% CI)	p-value
**Sex**					
Male	55.7 (230/413)	1			
Female	55.2 (159/288)	0.981 (0.725–1.327)	0.900		
**Age in months (categorized)**					
0–11	54.1 (158/292)	1			
12–23	54.2 (155/286)	1.004 (0.723–1.392)	0.983		
24–59	61.8 (76/123)	1.371 (0.892–2.109)	0.150		
**Province**					
Maputo city	47.3 (232/491)	1		1	
Sofala	72.9 (51/70)	2.997 (1.719–5.224)	< 0.001	2.045 (1.225–4.722)	0.011
Zambezia	71.4 (35/49)	2.791 (1.465–5.317)	0.002	4.072 (1.549–10.705)	0.004
Nampula	78.0 (71/91)	3.963 (2.340–6.712)	< 0.001	3.822 (2.085–7.007)	< 0.001
**Mother’s education level**					
None	89.4 (59/66)	1		1	
Primary	54.7 (150/274)	0.144 (0.063–0.325)	< 0.001	0.180 (0.070–0.462)	< 0.001
Secondary/above	50.1 (178/355)	0.119 (0.053–0.268)	< 0.001	0.162 (0.063–0.415)	< 0.001
Unknown	6				
**Exclusive breastfeeding**					
No	59.5 (310/521)	1		1	
Yes	44.2 (73/165)	0.540 (0.379–0.769)	< 0.001	0.704 (0.441–1.122)	0.140
Unknown	15				
**Year**					
2015	59.2 (45/76)	1			
2016	69.4 (75/108)	1.566 (0.847–2.893)	0.152		
2017	50.5 (109/216)	0.702 (0.413–1.192)	0.190		
2018	55.2 (96/174)	0.848 (0.491–1.464)	0.554		
2019	50.4 (64/127)	0.700 (0.394–1.243)	0.224		
**Low birth weight (< 2500 grams)**					
No	51.7 (274/530)	1		1	
Yes	72.8 (67/92)	2.504 (1.534–4.087)	< 0.001	2.420 (1.339–4.374)	0.003
Unknown	79				
**Child previously hospitalized due to diarrhea**					
No	53.1 (282/531)	1		1	
Yes	67.2 (45/67)	1.806 (1.055–3.092)	0.031	1.366 (0.717–2.599)	0.342
Unknown	103				
**Mother’s HIV status**					
No	47.0 (218/464)	1		1	
Yes	73.1 (141/193)	3.060 (2.121–4.415)	< 0.001	2.604 (1.657–4.091)	< 0.001
Unknown	44				

There were no significant differences between children with and without comorbidities by sex, age categorized and year (Table 2).

**Fig 3:**
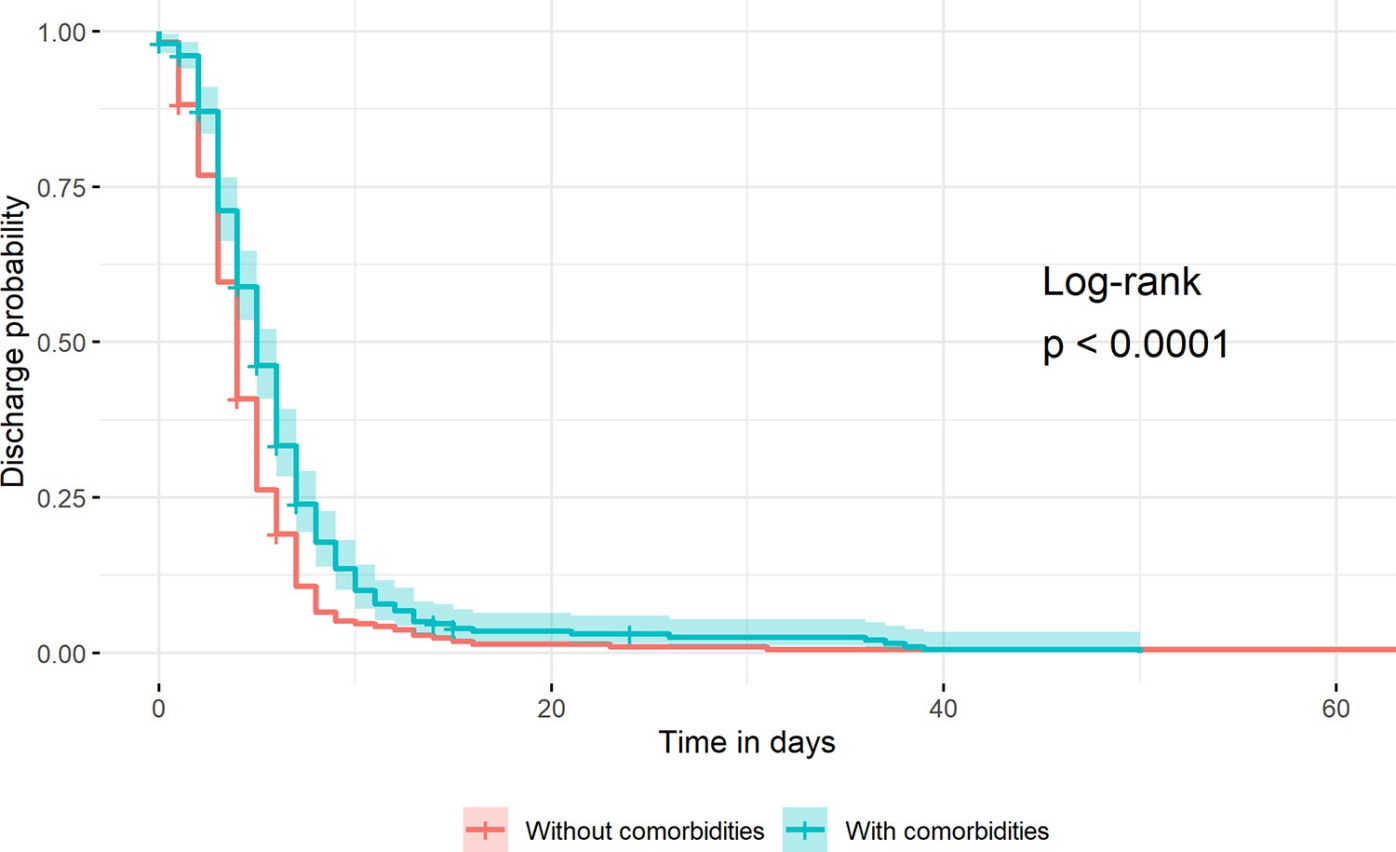
Discharge probability length by comorbidity status, January 2015 to December 2019.

Discharge probability was higher in children without comorbidities than in children with comorbidities (Fig 3). The median (interquartile range) length of hospital stay until discharge was significantly higher in children with comorbidities than those without comorbidities, 5 days (3–7) and 4 days (3–6) respectively (p-value < 0.001).

**Fig 4:**
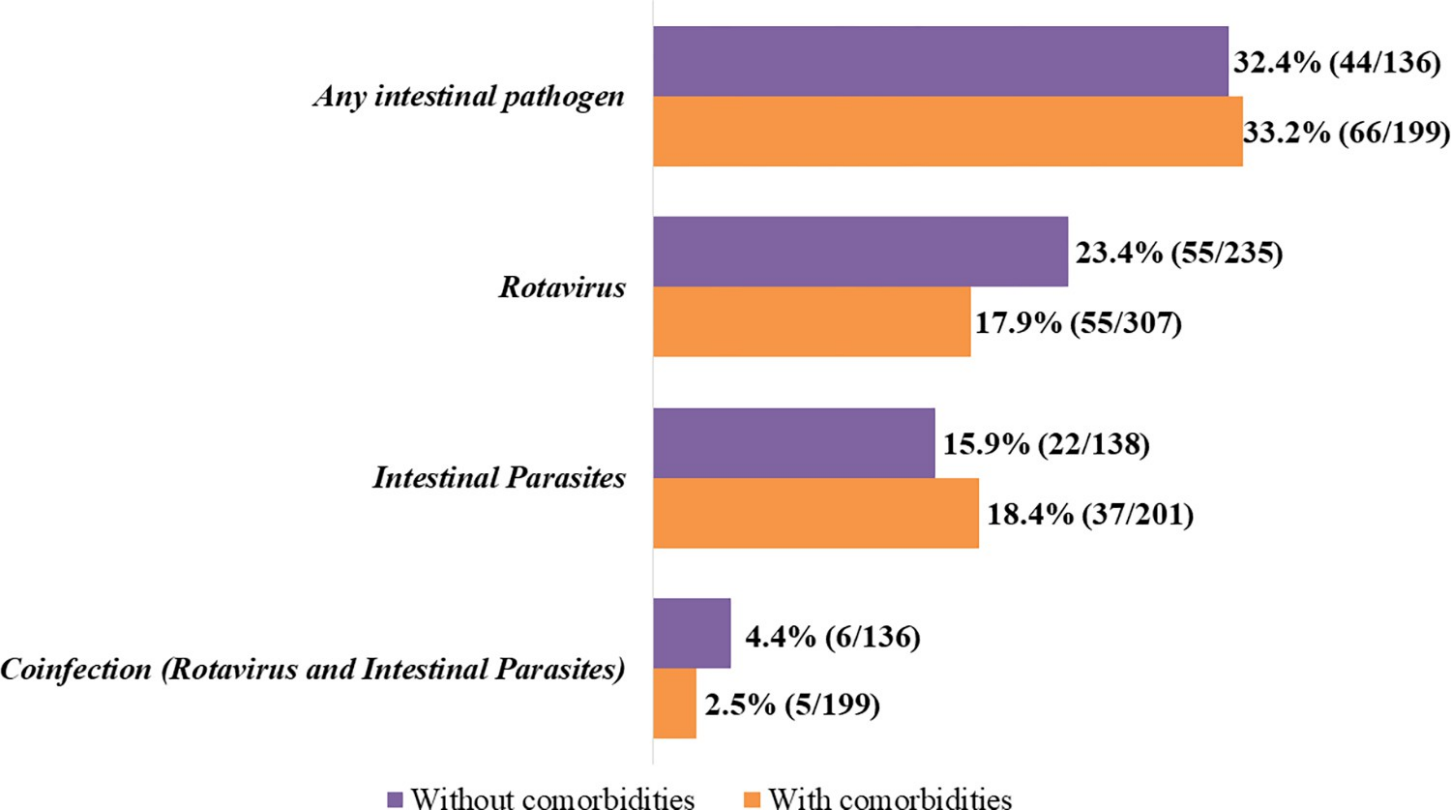
Intestinal pathogens by comorbidity status, January 2015 to December 2019.

Comorbidities did not increase the likelihood of having infection by any intestinal pathogen, Rotavirus A or intestinal parasite (p-value > 0.05, Fig 4).

## Discussion

In the present analysis, we observed that half of the Mozambican children up to 59 months of age seeking hospital care due to diarrhea in the six hospitals included in this analysis had an additional disease, such as wasting, stunting, HIV, malaria, or pneumonia. In other African countries, comorbidities in children up to 59 months of age ranged from 2% to 11% [6–10] using the Demographic Health Survey data, due to their advantage of representing the country’s population. Double occurrence of diarrhea and acute respiratory infections were observed in 11% of children in Ghana and 2% of children in Kenya [6, 8]. In Nigerian children, a reported 9% of diarrhea cases occurred simultaneously with acute respiratory infection and fever [10]. In South Africa, 64% of the children hospitalized with severe acute malnutrition also had diarrhea [22]. Differences in population eligibility criteria and settings (community versus hospital-based studies) may justify the proportions observed in our analysis compared to the reported literature.

Among the comorbidities, wasting and stunting were the most common. Overall Mozambique’s malnutrition estimates are around 40% [23, 24]. In children with diarrhea wasting and stunting have been observed in 27% and 33% respectively [3]. Additionally, it was also observed that malnutrition increases the likelihood of death in children with diarrhea in rural settings [5]. A higher frequency of malnutrition compared to the other identified diseases (HIV, malaria, pneumonia), may be due to the higher burden of malnutrition in the Mozambican population [23, 24]. Diarrhea and malnutrition are known to have a bi-directional association, diarrhea affecting the microbiota and impairing nutrient absorption and malnutrition as a proxy for immune deficiency which can lead to the appearance of clinical symptoms, such as diarrhea [25].

Comorbidities rates were higher in Hospital Central de Nampula and Hospital Central de Quelimane, located in the Nampula and Zambezia provinces, respectively. These two provinces have poorer health indicators, compared to Maputo city, located in the southern region of the country. Factors such as increased rates of malnutrition, high population density and lower rates of access to improved sanitation [23, 26], can contribute to the higher occurrence of comorbidities in Nampula and Zambezia hospital sites, mostly the ones associated with diarrhea and malnutrition. Nampula was the province with the highest rates of comorbidities in the present analysis. This province is the most populated in the country with the highest prevalence of chronic malnutrition (48.6%) which may explain the proportion observed [24].

Comorbidities were common in children whose mothers were illiterate. Education level is a proxy of socioeconomic status based on the hypothesis that the higher the caregiver’s education level, the lower the risk of an unfavorable health status [27, 28]. Literate caregiver may have additional knowledge and a better understanding of how to apply health recommendations, such as, boiling drinking water before giving it to the child to drink, as a measure to prevent diarrhea in their child.

In the crude logistics regression model, comorbidities were more common in children that didn’t receive exclusive breastfeeding. Breastfeed has a protective effect against diarrhea incidence, prevalence, duration, morbidity, and all-cause mortality [29]. In the sampled population, exclusive breastfeeding was done in 24% of the population, below the national prevalence of 43% [24], and below the global prevalence of 46% [30]. The global nutrition monitoring frameworks, target for 2025 is that at least 50% of children in the first six months receive exclusive breastfeeding [31]. Whether the proportion of children exclusively breastfed observed is subjected to population selection bias (i.e., only analyzing children seeking health care services due to diarrhea) or not, barriers and facilitators for exclusive breastfeeding prevalence should be assessed and promoted to increase the likelihood of achieving the 2025 target in children seeking health care services due to diarrhea.

HIV positive mothers were two times more likely to have a child with comorbidities, possibly because almost half of the children from the comorbidities group were positives for HIV S4 Table and Table 2.

Low birth weight was higher in children with comorbidities compared to children without comorbidities. It has been assessed that birth weight impacts a child’s development, the higher the weight, the better the cognition, receptive and language expression the child would present [32].

We observed an overall re-hospitalization above 10%. In the crude logistic regression model comorbidities were more common in children re-hospitalized than in children not re-hospitalized. Previous re-hospitalization was reported in children with diarrhea in Alexandria [33] and Kenya [34] in less than five percent of children. Re-hospitalizations are suggestive of incomplete treatment, indicating existing vulnerability even after discharge [34], which could be either because improper treatment that was provided to the identified child, or underdiagnosed underlying diseases that were not treated at all, during the child’s hospitalization.

A higher probability of hospital stay in children with comorbidities was observed, which may be related to additional complications, due to the need of the clinicians to manage additional health impairment (e.g.: HIV, malnutrition) until the child is stable for discharge [35].

This is a cross-sectional analysis, which cannot confirm causality between the comorbidity and independent characteristics. Self-reported information may have added bias to the data, thus, generalization should be done with caution. Upon discharge from the hospital, children’s survival in the community and risk of re-hospitalization were not assessed. Despite those limitations, we demonstrated the existence of comorbidities in children seeking health care for diarrhea in hospital sites in the three regions of Mozambique. Furthermore, we also observed that comorbidities can increase the length of hospital stay and the likelihood of re-hospitalization.

## Conclusions

Undernutrition was the most common comorbidity and was associated with birthweight. Policies advocating health education for pregnant women, including knowledge, attitudes, and practices should be considered, mainly in areas with low literacy levels, to reduce proportions of low birth weight and undernutrition. The integrated management of childhood illness strategy, introduced in the country should be effectively implemented. This is to ensure that all children with diarrhea also undergoes routine assessment of their nutritional status, facilitating early diagnosis and appropriate treatment. Health practitioners should implant and evaluate the feasibility and efficacy of the policies advocating health education mostly in illiterate pregnant women and their impact in reducing the comorbidities proportions, including individual occurrence of undernutrition, HIV, malaria, and pneumonia.

## Supporting information

S1 Checklist STROBE statement—checklist of items that should be included in reports of observational studies. (DOCX)

S1 Table Sociodemographic and clinical characteristics and factors associated with pneumonia in children with diarrhea, January 2015 to December 2019. (DOCX)

S2 Table Sociodemographic and clinical characteristics and factors associated with malaria in children with diarrhea, January 2015 to December 2019. (DOCX)

S3 Table Sociodemographic and clinical characteristics and factors associated with HIV in children with diarrhea, January 2015 to December 2019. (DOCX)

S4 Table Sociodemographic and clinical characteristics and factors associated with stunting/wasting in children with diarrhea, January 2015 to December 2019. (DOCX)
