# Supplementary material for: Examining comorbidities in children with diarrhea across four provinces of Mozambique: A cross-sectional study (2015 to 2019)
Source: PLoS One. 2023 Sep 26;18(9):e0292093. doi: 10.1371/journal.pone.0292093 (PMC10522033; doi:10.1371/journal.pone.0292093)
Supplement: S1 Table — (DOCX) [file pone.0292093.s002.docx]

**S1 Table. Sociodemographic and clinical characteristics and factors associated with pneumonia in children with diarrhea, January 2015 to December 2019.**

| **Characteristics** | **% (n/N)** | **COR (95% CI)** | **p-value** |
| --- | --- | --- | --- |
| **Sex** |  |  |  |
| Male | 1.7 (7/413) | 1 |  |
| Female | 1.7 (5/288) | 1.025 (0.322 - 3.261) | 0.967 |
| **Age in months (categorized)** |  |  |  |
| 0-11 | 2.7 (8/292) | 1 |  |
| 12-23 | 1.0 (3/286) | 0.376 (0.099 - 1.433) | 0.152 |
| 24-59 | 0.8 (1/123) | 0.291 (0.036 - 2.352) | 0.247 |
| **Province** |  |  |  |
| Maputo city | 1.8 (9/491) | 1 |  |
| Sofala | 2.9 (2/70) | 1.575 (0.333 - 7.444) | 0.566 |
| Zambezia | 0.0 (0/49) | - |  |
| Nampula | 1.1 (1/91) | 0.595 (0.074 - 4.755) | 0.624 |
| **Mother's education level** |  |  |  |
| None | 1.5 (1/66) | 1 |  |
| Primary | 2.2 (6/274) | 1.455 (0.172 - 12.298) | 0.730 |
| Secondary/above | 1.4 (5/355) | 0.929 (0.107 - 8.078) | 0.946 |
| Unknown | 6 |  |  |
| **Exclusive breastfeeding** |  |  |  |
| No | 1.5 (8/521) | 1 |  |
| Yes | 2.4 (4/165) | 1.593 (0.474 - 5.360) | 0.452 |
| Unknown | 15 |  |  |
| **Low birth weight**  **(< 2500 grams)** |  |  |  |
| No | 1.5 (8/530) | 1 |  |
| Yes | 2.2 (2/92) | 1.450 (0.303 - 6.939) | 0.642 |
| Unknown | 79 |  |  |
| **Child previously hospitalized due to diarrhea** |  |  |  |
| No | 1.9 (10/531) | 1 | 0.991 |
| Yes | 0.0 (0/67) | - |  |
| Unknown | 103 |  |  |
| **Mother's HIV status** |  |  |  |
| No | 0.9 (4/464) | 1 |  |
| Yes | 3.1 (6/193) | 3.690 (1.030 - 13.225) | 0.045 |
| Unknown | 44 |  |  |
