# Supplementary material for: Examining comorbidities in children with diarrhea across four provinces of Mozambique: A cross-sectional study (2015 to 2019)
Source: PLoS One. 2023 Sep 26;18(9):e0292093. doi: 10.1371/journal.pone.0292093 (PMC10522033; doi:10.1371/journal.pone.0292093)
Supplement: S2 Table — (DOCX) [file pone.0292093.s003.docx]

**S2 Table.** **Sociodemographic and clinical characteristics and factors associated with malaria in children with diarrhea, January 2015 to December 2019.**

| **Characteristics** | **% (n/N)** | **COR (95% CI)** | **p-value** | **AOR (95% CI)** | **p-value** |
| --- | --- | --- | --- | --- | --- |
| **Sex** |  |  |  |  |  |
| Male | 3.9 (16/413) | 1 |  |  |  |
| Female | 3.8 (11/288) | 0.985 (0.450 - 2.156) | 0.970 |  |  |
| **Age in months (categorized)** |  |  |  |  |  |
| 0-11 | 3.8 (11/292) | 1 |  |  |  |
| 12-23 | 2.8 (8/286) | 0.735 (0.291 - 1.855) | 0.515 |  |  |
| 24-59 | 6.5 (8/123) | 1.778 (0.697 - 4.532) | 0.229 |  |  |
| **Province** |  |  |  |  |  |
| Maputo city | 3.3 (16/491) | 1 |  | 1 |  |
| Sofala | 1.4 (1/70) | 0.430 (0.056 - 3.293) | 0.417 | 0.326 (0.041 - 2.613) | 0.292 |
| Zambezia | 10.2 (5/49) | 3.374 (1.180 - 9.646) | 0.023 | 2.992 (0.948 - 9.441) | 0.062 |
| Nampula | 5.5 (5/91) | 1.726 (0.616 - 4.835) | 0.299 | 2.074 (0.636 - 6.762) | 0.226 |
| **Mother's education level** |  |  |  |  |  |
| None | 9.1 (6/66) | 1 |  | 1 |  |
| Primary | 4.4 (12/274) | 0.458 (0.165 - 1.269) | 0.133 | 0.479 (0.166 - 1.382) | 0.173 |
| Secondary/above | 2.5 (9/355) | 0.260 (0.089 - 0.757) | 0.014 | 0.287 (0.096 - 0.861) | 0.026 |
| Unknown | 6 |  |  |  |  |
| **Exclusive breastfeeding** |  |  |  |  |  |
| No | 4.0 (21/521) | 1 |  |  |  |
| Yes | 3.0 (5/165) | 0.744 (0.276 - 2.005) | 0.559 |  |  |
| Unknown | 15 |  |  |  |  |
| **Year** |  |  |  |  |  |
| 2015 | 9.2 (7/76) | 1 |  | 1 |  |
| 2016 | 5.6 (6/108) | 0.580 (0.187 - 1.799) | 0.346 | 0.441 (0.127 - 1.529) | 0.197 |
| 2017 | 0.9 (2/216) | 0.092 (0.019 - 0.454) | 0.003 | 0.094 (0.019 - 0.477) | 0.004 |
| 2018 | 4.6 (8/174) | 0.475 (0.166 - 1.361) | 0.166 | 0.513 (0.175 - 1.507) | 0.225 |
| 2019 | 3.1 (4/127) | 0.321 (0.091 - 1.134) | 0.078 | 0.225 (0.056 - 0.907) | 0.036 |
| **Low birth weight**  **(< 2500 grams)** |  |  |  |  |  |
| No | 3.4 (18/530) | 1 |  |  |  |
| Yes | 4.3 (4/92) | 1.293 (0.427 - 3.911) | 0.649 |  |  |
| Unknown | 79 |  |  |  |  |
| **Child previously hospitalized due to diarrhea** |  |  |  |  |  |
| No | 4.1 (22/531) | 1 |  |  |  |
| Yes | 3.0 (2/67) | 0.712 (0.164 - 3.097) | 0.651 |  |  |
| Unknown | 103 |  |  |  |  |
| **Mother's HIV status** |  |  |  |  |  |
| No | 3.7 (17/464) | 1 |  |  |  |
| Yes | 3.1 (6/193) | 0.844 (0.328 - 2.173) | 0.725 |  |  |
| Unknown | 44 |  |  |  |  |
