# Supplementary material for: Examining comorbidities in children with diarrhea across four provinces of Mozambique: A cross-sectional study (2015 to 2019)
Source: PLoS One. 2023 Sep 26;18(9):e0292093. doi: 10.1371/journal.pone.0292093 (PMC10522033; doi:10.1371/journal.pone.0292093)
Supplement: S3 Table — (DOCX) [file pone.0292093.s004.docx]

**S3 Table. Sociodemographic and clinical characteristics and factors associated with HIV in children with diarrhea, January 2015 to December 2019.**

| **Characteristics** | **% (n/N)** | **COR (95% CI)** | **p-value** | **AOR (95% CI)** | **p-value** |
| --- | --- | --- | --- | --- | --- |
| **Sex** |  |  |  |  |  |
| Male | 12.3 (51/413) | 1 |  | 1 |  |
| Female | 8.0 (23/288) | 0.616 (0.367 - 1.033) | 0.066 | 0.551 (0.274 - 1.107) | 0.094 |
| **Age in months (categorized)** |  |  |  |  |  |
| 0-11 | 8.6 (25/292) | 1 |  | 1 |  |
| 12-23 | 13.6 (39/286) | 1.686 (0.991 - 2.868) | 0.054 | 1.878 (0.910 - 3.873) | 0.088 |
| 24-59 | 8.1 (10/123) | 0.945 (0.440 - 2.032) | 0.885 | 1.632 (0.543 - 4.901) | 0.383 |
| **Province** |  |  |  |  |  |
| Maputo city | 9.6 (47/491) | 1 |  |  |  |
| Sofala | 12.9 (9/70) | 1.394 (0.651 - 2.985) | 0.393 |  |  |
| Zambezia | 12.2 (6/49) | 1.318 (0.533 - 3.260) | 0.550 |  |  |
| Nampula | 13.2 (12/91) | 1.435 (0.729 - 2.826) | 0.296 |  |  |
| **Mother's education level** |  |  |  |  |  |
| None | 28.8 (19/66) | 1 |  | 1 |  |
| Primary | 9.5 (26/274) | 0.259 (0.133 - 0.506) | < 0.001 | 0.360 (0.134 - 0.966) | 0.043 |
| Secondary/above | 7.9 (28/355) | 0.212 (0.110 - 0.409) | < 0.001 | 0.447 (0.167 - 1.196) | 0.109 |
| Unknown | 6 |  |  |  |  |
| **Exclusive breastfeeding** |  |  |  |  |  |
| No | 11.9 (62/521) | 1 |  | 1 |  |
| Yes | 6.1 (10/165) | 0.478 (0.239 - 0.954) | 0.036 | 0.733 (0.291 - 1.849) | 0.511 |
| Unknown | 15 |  |  |  |  |
| **Year** |  |  |  |  |  |
| 2015 | 11.8 (9/76) | 1 |  |  |  |
| 2016 | 13.9 (15/108) | 1.201 (0.496 - 2.907) | 0.685 |  |  |
| 2017 | 10.2 (22/216) | 0.844 (0.370 - 1.924) | 0.687 |  |  |
| 2018 | 12.1 (21/174) | 1.022 (0.445 - 2.348) | 0.960 |  |  |
| 2019 | 5.5 (7/127) | 0.434 (0.155 - 1.219) | 0.113 |  |  |
| **Low birth weight**  **(< 2500 grams)** |  |  |  |  |  |
| No | 10.8 (57/530) | 1 |  |  |  |
| Yes | 8.7 (8/92) | 0.790 (0.364 - 1.716) | 0.552 |  |  |
| Unknown | 79 |  |  |  |  |
| **Child previously hospitalized due to diarrhea** |  |  |  |  |  |
| No | 8.9 (47/531) | 1 |  | 1 |  |
| Yes | 16.4 (11/67) | 2.023 (0.992 - 4.124) | 0.053 | 1.426 (0.592 - 3.433) | 0.429 |
| Unknown | 103 |  |  |  |  |
| **Mother's HIV status** |  |  |  |  |  |
| No | 0.6 (3/464) | 1 |  | 1 |  |
| Yes | 35.2 (68/193) | 83.595 (25.871 - 270.109) | < 0.001 | 59.221 (17.814 - 196.881) | < 0.001 |
| Unknown | 44 |  |  |  |  |
