# Supplementary material for: Examining comorbidities in children with diarrhea across four provinces of Mozambique: A cross-sectional study (2015 to 2019)
Source: PLoS One. 2023 Sep 26;18(9):e0292093. doi: 10.1371/journal.pone.0292093 (PMC10522033; doi:10.1371/journal.pone.0292093)
Supplement: S4 Table — (DOCX) [file pone.0292093.s005.docx]

**S4 Table. Sociodemographic and clinical characteristics and factors associated with stunting/wasting in children with diarrhea, January 2015 to December 2019.**

| **Characteristics** | **% (n/N)** | **COR (95% CI)** | **p-value** | **AOR (95% CI)** | **p-value** |
| --- | --- | --- | --- | --- | --- |
| **Sex** |  |  |  |  |  |
| Male | 51.3 (212/413) | 1 |  |  |  |
| Female | 51.0 (147/288) | 0.988 (0.732 - 1.336) | 0.940 |  |  |
| **Age in months (categorized)** |  |  |  |  |  |
| 0-11 | 49.7 (145/292) | 1 |  |  |  |
| 12-23 | 49.7 (142/286) | 1.000 (0.722 - 1.385) | 0.999 |  |  |
| 24-59 | 58.5 (72/123) | 1.431 (0.935 - 2.191) | 0.100 |  |  |
| **Province** |  |  |  |  |  |
| Maputo city | 42.2 (207/491) | 1 |  | 1 |  |
| Sofala | 71.4 (50/70) | 3.430 (1.982 - 5.937) | < 0.001 | 3.006 (1.539 - 5.872) | 0.001 |
| Zambezia | 67.3 (33/49) | 2.830 (1.517 - 5.278) | 0.001 | 4.290 (1.726 - 10.665) | 0.002 |
| Nampula | 75.8 (69/91) | 4.303 (2.578 - 7.182) | < 0.001 | 4.146 (2.310 - 7.442) | < 0.001 |
| **Mother's education level** |  |  |  |  |  |
| None | 81.8 (54/66) | 1 |  | 1 |  |
| Primary | 49.6 (136/274) | 0.219 (0.112 - 0.427) | < 0.001 | 0.272 (0.121 -0.608) | 0.002 |
| Secondary/above | 47.0 (167/355) | 0.197 (0.102 - 0.382) | < 0.001 | 0.270 (0.121 - 0.603) | 0.001 |
| Unknown | 6 |  |  |  |  |
| **Exclusive breastfeeding** |  |  |  |  |  |
| No | 55.1 (287/521) | 1 |  | 1 |  |
| Yes | 40.6 (67/165) | 0.557 (0.391 - 0.795) | 0.001 | 0.714 (0.448 - 1.139) | 0.157 |
| Unknown | 15 |  |  |  |  |
| **Year** |  |  |  |  |  |
| 2015 | 55.3 (42/76) | 1 |  |  |  |
| 2016 | 63.9 (69/108) | 1.432 (0.787 - 2.607) | 0.240 |  |  |
| 2017 | 47.2 (102/216) | 0.724 (0.428 - 1.225) | 0.229 |  |  |
| 2018 | 48.9 (85/174) | 0.773 (0.450 - 1.328) | 0.351 |  |  |
| 2019 | 48.0 (61/127) | 0.748 (0.423 - 1.324) | 0.319 |  |  |
| **Low birth weight**  **(< 2500 grams)** |  |  |  |  |  |
| No | 47.2 (250/530) | 1 |  | 1 |  |
| Yes | 69.6 (64/92) | 2.560 (1.591 - 4.119) | < 0.001 | 2.330 (1.312 - 4.140) | 0.004 |
| Unknown | 79 |  |  |  |  |
| **Child previously hospitalized due to diarrhea** |  |  |  |  |  |
| No | 48.8 (259/531) | 1 |  | 1 |  |
| Yes | 65.7 (44/67) | 2.009 (1.180 - 3.421) | 0.010 | 1.666 (0.888 - 3.128) | 0.112 |
| Unknown | 103 |  |  |  |  |
| **Mother's HIV status** |  |  |  |  |  |
| No | 44.2 (205/464) | 1 |  | 1 |  |
| Yes | 64.2 (124/193) | 2.270 (1.605 - 3.211) | < 0.001 | 2.219 (1.431 - 3.441) | < 0.001 |
| Unknown | 44 |  |  |  |  |
